# Adjusting Phenotypes by Noise Control

**DOI:** 10.1371/journal.pcbi.1002344

**Published:** 2012-01-12

**Authors:** Kyung H. Kim, Herbert M. Sauro

**Affiliations:** Department of Bioengineering, University of Washington, Seattle, Washington, United States of America; Johns Hopkins University, United States of America

## Abstract

Genetically identical cells can show phenotypic variability. This is often caused by stochastic events that originate from randomness in biochemical processes involving in gene expression and other extrinsic cellular processes. From an engineering perspective, there have been efforts focused on theory and experiments to control noise levels by perturbing and replacing gene network components. However, systematic methods for noise control are lacking mainly due to the intractable mathematical structure of noise propagation through reaction networks. Here, we provide a numerical analysis method by quantifying the parametric sensitivity of noise characteristics at the level of the linear noise approximation. Our analysis is readily applicable to various types of noise control and to different types of system; for example, we can orthogonally control the mean and noise levels and can control system dynamics such as noisy oscillations. As an illustration we applied our method to HIV and yeast gene expression systems and metabolic networks. The oscillatory signal control was applied to p53 oscillations from DNA damage. Furthermore, we showed that the efficiency of orthogonal control can be enhanced by applying extrinsic noise and feedback. Our noise control analysis can be applied to any stochastic model belonging to continuous time Markovian systems such as biological and chemical reaction systems, and even computer and social networks. We anticipate the proposed analysis to be a useful tool for designing and controlling synthetic gene networks.

## Author Summary

Stochastic gene expression at the single cell level can lead to significant phenotypic variation at the population level. To obtain a desired phenotype, the noise levels of intracellular protein concentrations may need to be tuned and controlled. Noise levels often decrease in relative amount as the mean values increase. This implies that the noise levels can be passively controlled by changing the mean values. In an engineering perspective, the noise levels can be further controlled while the mean values can be simultaneously adjusted to desired values. Here, systematic schemes for such simultaneous control are described by identifying where and by how much the system needs to be perturbed. The schemes can be applied to the design process of a potential therapeutic HIV-drug that targets a certain set of reactions that are identified by the proposed analysis, to prevent stochastic transition to the lytic state. In some cases, the simultaneous control cannot be performed efficiently, when the noise levels strongly change with the mean values. This problem is shown to be resolved by applying extra noise and feedback.

## Introduction

There have been numerous experiments conducted on a wide range of organisms such as prokaryotic [1]–[3] and eukaryotic [4], [5] cells including mammalian cells [6], [7], to study gene expression noise. The noise originates from randomness in biochemical processes involving in transcription-translation, shared synthesis-degradation mechanisms [8], the cell cycle [9], [10], and other unidentified processes. Stochastic gene expression can lead to significant phenotypic cell-to-cell variation. For example, the stochasticity can help cells survive in stress environment [11]–[13] or determine the fate of viruses between latency and reactivation by randomly switching the two states [14], [15]. In metabolic networks, noise in enzyme levels causes metabolic flux to fluctuate and eventually can reduce the growth rate of host cells [16].

Although the measured noise is often explained by mathematical models [1]–[7], a systematic analysis on parametric control of noise has been lacking. This is attributed to the fact that noise propagation through pathway connections generates correlations between the pathway species [17], which make analysis difficult. Most noise control analyses have been focused on identifying the analytical structure of the noise propagation [17]–[19]. As the system size increases, the mathematical structure, however, becomes highly intractable. There have been some efforts to describe noise propagation in a modular way [18]. However, complicated feedback and feedforward structures in real biological networks hamper modular noise analysis.

Here, we are concerned with control of noise in biological systems such as gene regulatory networks and metabolic networks. In particular, we are interested in independent (orthogonal) control of noise and mean levels. For example, noise can stochastically switch one gene expression state to another via stochastic switching. This phenomenon was investigated in the expression of ComK that regulates DNA uptake in *Bacillus subtilis* [12]. The study used orthogonal control of noise to show that the reduction in the expression noise decreases the switching to competence [12]. Similarly, one can study how stochastic viral decisions [15] are made by independently changing the noise and mean levels of viral gene expression. Their individual contributions can be compared and used for identifying noise control schemes. This could eventually provide an efficient way to prevent viral activation. Here, we provide a systematic mathematical analysis method for simultaneous control of noise and mean levels and apply it to a number of well known biological examples.

We approach this control problem numerically by quantifying the parametric sensitivity of noise characteristics at the level of the linear noise approximation [20]. Our numerical approach, which we name stochastic control analysis [21], is practical in interpreting noise control experiments and computationally efficient and scalable in system size. Based on our analysis method, ‘active’ control of noise is proposed to manipulate the noise. We pursue various control schemes, such as independent control of mean and noise levels (such control will be called orthogonal), control of multiple mean and noise levels with certain ratios, and control of system dynamics of noisy oscillations. Active noise control can be applied to modify natural gene regulatory networks and improve their noise-related phenotype, and furthermore to design and construct gene regulatory networks for better performance by exploiting noise. It can be applied not only to gene regulatory networks but also to other biological systems such as metabolic networks [16].

In addition, we make a connection between noise control and network structure, and propose the mechanisms that could enhance the efficiency of orthogonal control. In a certain class of metabolic networks [22], probability distribution functions of each metabolite concentration were shown to be statistically independent of other species at the stationary state. The same result was also found in zero-range processes [23] in physics, complex balanced systems [24] and Jackson queueing networks [25] in mathematics. This independence was shown to be rooted to a certain network structure satisfying Feinberg's deficiency zero theorem [24]–[26]. We will show that when such species independence occurs, the orthogonal control of mean and noise levels is not possible, but that the application of extrinsic noise or feedback could help achieve orthogonal control.

## Results

### Stochastic sensitivity

For the purpose of noise control, we introduce stochastic sensitivities [21] called control coefficients (CCs) similar to the control coefficients in metabolic control analysis (MCA) [27]–[29]. These coefficients quantify the response of a system (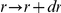) from one stationary state to another due to a parameter perturbation (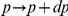), mathematically defined by
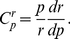
(1)The system parameters can include reaction rate constants [21], and the system responses include the mean and noise levels of concentrations and the temporal correlations of the concentrations (i.e., autocorrelations [30]).

CCs have been widely used in MCA for metabolic networks in the deterministic framework [27]–[29]. Here we use CCs to control noise in stochastic systems [3], [21]. Since noise can be considered a response of continuous perturbations in system parameters, the attributes of the dynamical response of the system (such as the period and amplitude of oscillations) [2], [3], [6], [31]–[34] can be deduced from noise characteristics, such as autocorrelations [30]. Thus, stochastic CCs also can be used to control system dynamics.

The noise level is defined as variance (covariance) divided by mean square (product of two mean values). We compute the noise levels and auto-correlations at the first level of approximation (see Methods) such that the noise level is assumed to be small enough that the rate laws can be linearized. From the computed noise levels and auto-correlations, we obtain the CCs (see Methods) to indicate where and by how much the system parameters are controlled.

### Control vector

In deterministic classical control theory [35] and MCA [36]–[39], the orthogonal control of system variables (flux and concentrations) has been studied. Here, we mainly consider orthogonal control in the stochastic regime to independently control mean and noise levels of concentrations. The noise level is often strongly anti-correlated with its mean level; for example, when a molecular species degrades with a first order reaction and is synthesized at a constant rate, the concentration level follows the Poisson distribution, where the variance is equal to the mean value, i.e., the noise level is equal to the inverse of the mean value. Thus, orthogonal noise control typically requires two or more parameters to be perturbed. In addition, the noise level shows non-local correlations between different species of molecules due to noise propagation [17], [40]. This also implies that a set of multiple parameters may need to be controlled simultaneously. Taking into account these points, we present a systematic non-local method for orthogonal control using the control coefficients.

We introduce a control vector
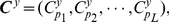
(2)which is defined in an 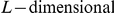 control parameter space. By the definition of the control coefficients, the inner product between 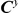 and a parameter perturbation vector gives the change in the system variable 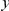 due to the perturbation:
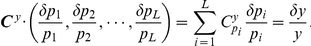
By denoting the parameter perturbation vector by 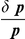, the above equation becomes:
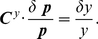
(3)When parameters 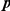 are perturbed in the **direction** of 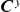, a system variable 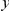 (concentration mean or noise level) will **increase**. When 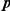 are perturbed in one of the **perpendicular** directions to 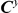, the system variable 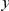 does **not change** (one particular direction is 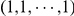. This corresponds to MCA-like summation theorems [21]).

For example, consider the following synthesis-degradation process:
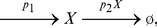
(4)where 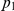 is a constant synthesis rate and 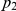 a degradation rate constant. These two parameters are considered the control parameters: 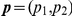. We aim to reduce the noise level of 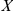 without changing its mean level. At the stationary state, the mean synthesis rate equals the mean degradation rate: 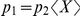. Therefore, the mean level at the stationary state becomes
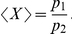
The noise level becomes 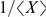, since the probability distribution of 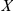 satisfies the Poisson distribution function and one of its properties is that the variance of 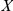 is equal to the mean level of 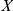. Therefore, the noise level can be obtained as
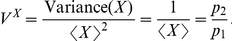
The control vectors for the mean and noise level can be calculated by using the definition of CCs, Eq. (1):
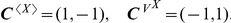
When the parameters are perturbed in the perpendicular direction of 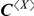:
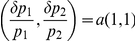
with 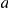 a non-zero real number, the mean level does not change [21]. However, since the noise level is the inverse of the mean value, the noise level does not change, either [21]. This is because the control vector for the noise level is anti-parallel with that of the mean value. Therefore, when a species concentration satisfies the Poisson distribution function, its orthogonal control is impossible. The appearance of the Poisson distribution is known to be generalized for a certain class of mass-action networks that satisfy complex balance [24]. We will show later that the application of extrinsic noise and feedback onto these networks enable orthogonal control.

### Generalized control

In the last section, we saw a simple system, where we could not achieve orthogonal control. This begs the question, what networks can be controlled. This section describes how to answer this question and in addition, if controllable, how to determine the direction of parameter perturbations.

Consider that the vector of system variables 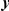, represented by 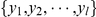, that is to be changed by percentage amounts 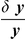 via parameter perturbations 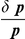. Once control coefficients are computed, the parameter perturbations 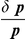 can be obtained by solving Eq. (3). The unit vector of 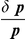, denoted by 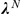, indicates the **direction of control**.

In the case of orthogonal control considered in the system (4), the mean level of 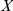 (denoted by 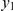) was aimed to be fixed, and its noise level (denoted by 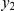) to be decreased, here for example by 3% 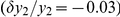. These system variables were controlled by perturbing 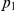 and 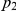. Thus, Eq. (3) can be written in the following matrix form:
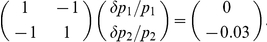
This equation has no solution for 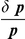, meaning that the desired control cannot be achieved and is overly-constrained. When the desired control is given by 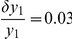 and 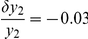 (not an orthogonal control case), the control can be, however, achieved in various ways. Eq. (3) becomes simplified to 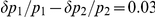. There are infinite number of solutions and Eq. (3) is then called degenerate.

In degenerate cases, we need to determine the direction of control that requires the minimum amount of change in system parameters for a given change in system variables. Mathematically, Eq. (3) can be solved for 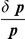, where the norm (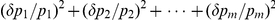) is minimized, by using the Lagrange multiplier method (see the Methods). 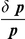 is normalized to obtain the direction of control 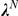.

### Orthogonal control

This section focuses on orthogonal control between two system variables, noise level 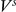 and mean value 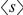. We aim to reduce the concentration noise level with its mean level fixed.

#### Control direction

Although the direction of the orthogonal control, 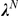, can be obtained by using the Langrange multiplier method (see Methods) as described in the previous section, we describe the following equivalent way of finding it to help understand the physical meaning of 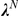.

**Figure 1:**
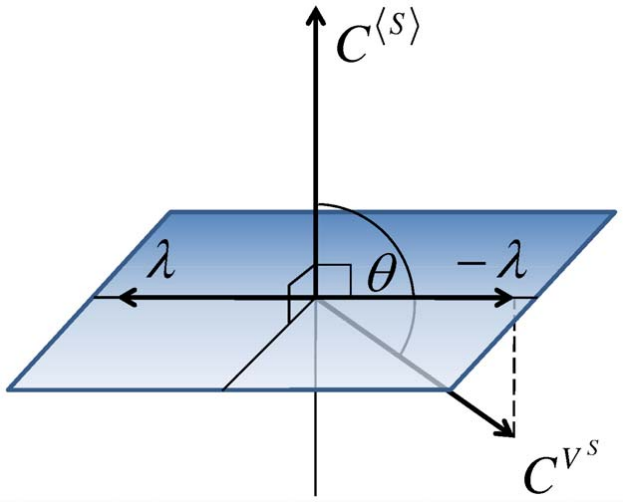
Control vector analysis for noise reduction. The noise level of a concentration (variance divided by mean squared) is aimed to be reduced while its mean level does not change. When parameter perturbations 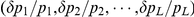 are performed within the space perpendicular to the control vector 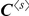 for the mean level, the mean concentration does not change. A control vector 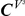 for the concentration noise level is projected onto the perpendicular space. The projected vector is denoted by 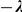. When parameter perturbations are directed along 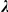 (the opposite direction of 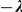), the noise level will decrease while the mean concentration does not change.

1. Compute a control vector for 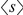.
2. Find the perpendicular space to 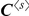. All the parameter perturbations within the perpendicular space do not change 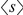 (Fig. 1).
3. Compute a control vector for the concentration noise level, 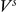.
4. Project the control vector for 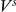 onto the perpendicular space and multiply the projected vector by 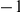. The resultant vector is denoted by 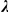 (Fig. 1) and its normalized form by 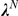.

The vector 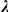 can be mathematically expressed as
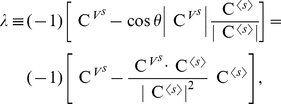
(5)where the factor of 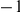 makes the noise level decrease and 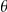 is the angle between the two control vectors 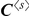 and 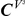 (Fig. 1). The unit vector of this 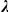 is shown to be identical to that of 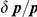 obtained via the Lagrangian multiplier method (see the Methods). The vector 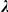 will be named the **orthogonal-control vector**.

#### Control strength and efficiency

We define the **strength** (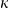) of this orthogonal control as the norm of the orthogonal-control vector:
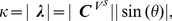
(6)where the term 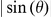 quantifies how much percentage ratio of the control vector for noise level is projected onto the perpendicular space. This defines the efficiency of the orthogonal control:
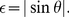
(7)Thus, the control strength is related to the efficiency as
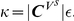
The higher efficiency leads to the higher control strength. If 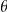 is close to 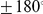, the two controls are anti-correlated and 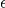 and 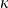 are 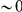. If 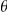 is close to 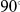, the two controls are already orthogonal and 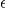 is 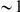 and the maximum control strength can be reached: 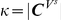. Therefore, the most efficient orthogonal control is achieved when the two control vectors are perpendicular, i.e., 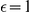 and the orthogonal control is not possible when 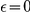.

Under experimental conditions, not all system parameters can be controlled. Thus, it is more appropriate for a control vector to be defined in a subspace of the full parameter space. For example, 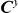 can represent 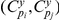 for two-parameter controls, where 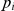 and 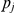 are chosen for perturbation and the other parameters are held constant. All the proposed quantities characterizing orthogonal controls such as 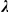, 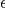, and 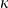 can be applied to the control vector defined in the subspace. Let us now consider specific examples of orthogonal control in other systems.

### Orthogonal control between noise and mean levels

**Figure 2:**
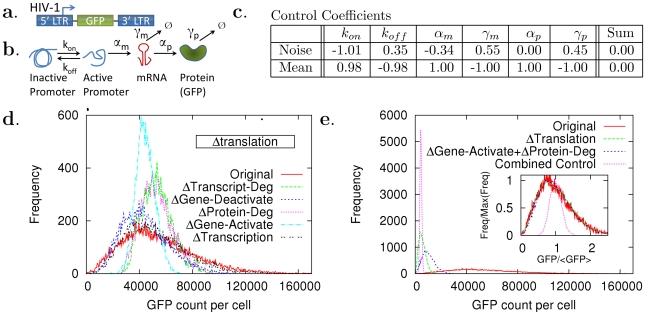
Orthogonal control of mean and noise levels in the HIV-1 LTR-promoter expression. (a) The HIV-1 model vector with a green fluorescence protein (GFP) gene that is transfected to Jurkat cells [42] is considered. (b) The promoter inactive and active states are explicitly represented by the two-state model [4], [41]. Based on the values of the control coefficients (provided in (c)), *in silico* perturbation experiments were designed. (d) The noise level was reduced without changing its mean level. The translation rate was decreased 10 times and one of the reactions among transcript degradation, gene deactivation, and protein degradation was decreased 10 times, or one of the reactions among gene activation and transcription was increased 10 times. (e) The mean level was reduced without changing the noise level either by decreasing the translation 10 times (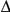
Translation), or by increasing the gene activation by twice and protein degradation 10 times (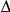
Gene-Activate + 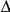
Protein-Deg). Two orthogonal control schemes were combined so that both the noise and mean levels were simultaneously controlled. The combined control was performed by decreasing the translation rate 100 times and increasing the gene activation 10 times (Combined Control).

We consider single-promoter gene expression systems to show orthogonal control of noise and mean expression levels. Yeast promoter GAL10 [11], [41] and HIV-1 long terminal repeat (LTR) promoter [42] show significant gene expression noise that mainly originates from transcriptional bursting [11], [42]: Once chromatin structure is remodeled, RNA polymerase II enzymes, while waiting for the remodeling, can continue the transcription elongation process in a bursting manner [4], [11], [42]–[44]. This phenomenon has been modeled as a two-state model describing stochastic gene activation and deactivation [4], [42], [44] (cf. [11] and see Fig. 2b):
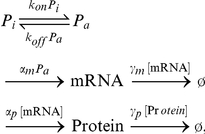
where 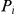 and 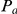 denote inactive and active states of a promoter and the functions that are placed above or below the arrows are reaction rates, not constants.

Here we identify which parameter control scheme is optimal for noise and mean level orthogonal control. We constrain ourselves to the case that two parameters can be controlled for each experiment. For all possible two-parameter combinations, control efficiency and strength are computed, and the parameter combination leading to the best efficiency and strength is identified as the most optimal control scheme.

#### HIV-1 LTR promoters

The HIV-1 long terminal repeat (LTR) promoter shows significant gene expression noise that mainly originates from transcriptional bursting [42]. We aim to identify control schemes that independently changes the mean and noise levels of the LTR promoter expression. The identified schemes will be combined to provide simultaneous control of both noise and mean, and this can be useful for a potential application to viral latency decision by preventing stochastic switching from the low basal expression state to the high trans-activated state [45].

**Figure 3:**
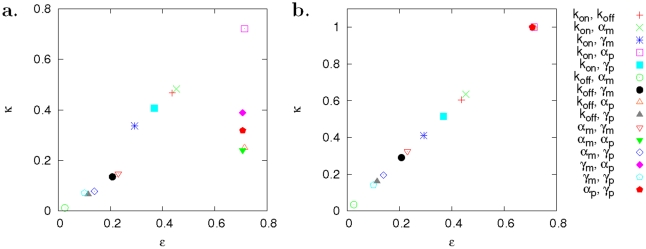
Efficiency and strength of orthogonal control in the HIV-1 expression system (**Fig. 2a**). All possible two-parameter controls were considered. The efficiency 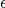 and strength 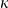 were computed by using Eqs. (6) and (7). (a) Among noise reduction control schemes, the most efficient and strongest one was related to gene activation 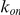 and translation 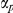. (b) Among mean-level reduction controls, the most efficient and strongest control schemes were related to translation 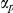 (collapsed data points).

The two-state model proposed in [42] was investigated (Fig. 2b). The total number of the promoter (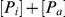) was assumed to be 1, and 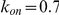, 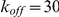, 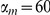, 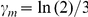, 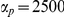, 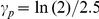 with the unit of all the parameters 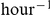, where the number of molecule is considered unitless. We devised two-parameter control schemes to reduce the noise level of the LTR promoter expression without affecting the mean level. Consider one specific set of parameters for perturbation: gene activation 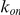 and translation 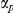 (this set will lead to the most sensitive control). The corresponding control vectors were found to be
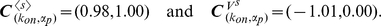
From Eq. (5), the orthogonal-control vector was obtained by
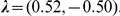
The strength and efficiency of the control was obtained from Eqs. (6) and (7), respectively:
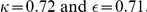


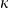
 and 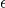 being close to one means that quite strong control can be achieved with high efficiency. We have performed all possible two-parameter control analysis. Most efficient controls were found to be related to 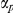 and among them, the one related to 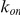 was strongest (Fig. 3a).

Why are control schemes that are related to 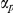 most efficient? Based on the computed CCs, decreasing translation reduces the mean level while the noise level does not change. All other reactions, however, can make the noise level to decrease although the mean level increases [42]–[44]. This indicates that the noise level can be reduced efficiently without affecting its mean level by perturbing 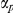 and one of the other reactions simultaneously.

Experimentally, this type of control is plausible. The translation 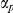 can be controlled by mutating the internal ribosome entry site (IRES) [46] or controlling translation initiation factors. The gene activation 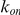 can be induced by tumor necrosis factor-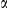, causing the noise level to decrease while the product of the noise and mean levels stays the same [42] (this is reflected in the CCs of 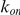 for noise and mean levels as being approximately -1 and 1, respectively, in Fig. 2c, second column.)

We can also devise another type of control such as orthogonal control of the mean level reduction. We examined all two-parameter control schemes by computing 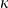 and 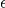, and found that the most efficient and strongest control is achieved by decreasing the translation rate without perturbing other reactions (the data points related to the translation were collapsed in Fig. 3b). The second best controls were found to be related to gene activation 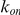. For example, we could achieve the mean level control by increasing gene activation 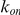 and protein degradation 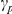 together, where the control for each parameter will compensate the mean-level change that would have occurred by the other control.

Stochastic simulations [47] were performed to verify the proposed orthogonal control methods and successfully showed that the noise/mean level was significantly reduced without changing the mean/noise level (Fig. 2d and e). This result shows that the reduction of translation activity 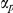 is very important for both the noise and mean level reduction.

We combined the two orthogonal control schemes that change the noise and mean levels independently. The combined control, by perturbing 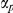 and 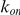, showed significant amounts of reduction in both the mean and noise levels (Fig. 2e). This provides efficient mechanisms for preventing stochastic switching to the high trans-activated state via simultaneous control on the mean and noise levels, and can be useful for a potential HIV drug design by preventing stochastic switching from the low basal expression state to the high trans-activated state.

**Figure 4:**
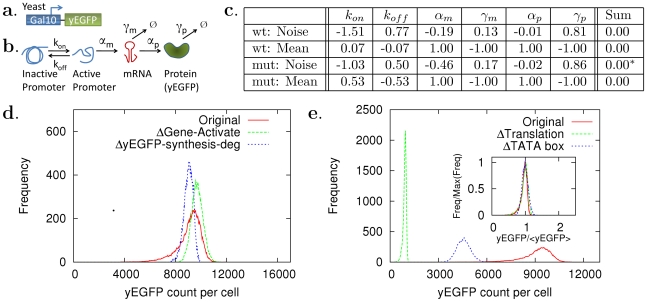
Orthogonal control of mean and noise levels in the Gal10 promoter expression. (a) The yeast Gal10 promoter, expressing yeast-enhanced green fluorescent protein (yEGFP) [41], is considered and (b) mathematically described with the two-state model. (c) Control coefficients were computed for the wild-type and TATA-box mutated promoters. Based on the values of the control coefficients, *in silico* perturbation experiments were designed. (d) The noise level was reduced without changing its mean level. 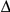
Gene-Activate: 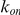 was increased 10 times. 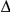
yEGFP-synthesis-deg: 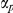 and 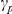 were decreased 10 times. (e) The mean level was reduced without changing the noise level either by decreasing 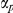 10 times (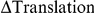), or by increasing 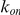 15 times and 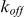 225 times (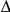
TATA box). 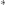: The actual sum is zero, but the sum of the round-up control coefficient values (shown in (c)) is 0.02 due to a round-up error.

We note that the CCs for noise levels show an interesting relationship among themselves. The CCs sum up to zero as shown in Fig. 2c and 4c. It can be theoretically proved that there exist summation theorems (similar to those found in MCA [27]–[29]) for the CCs for noise levels (second moment) and even higher moments [21]. The theorems directly indicate that the sensitivities are correlated with one another in a nontrivial way.

#### Yeast promoters

For the yeast promoter GAL10, it was shown that the mean level of promoter expression changes without altering the noise level under TATA box mutations [41]. The mutations were known to strongly affect yeast promoters such as GAL10 and GAL1 by increasing their promoter deactivation rate (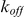) with a smaller effect on promoter activation (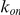) [11], [41], [48] but no effect on the transcription rate. However, for another yeast promoter PHO5, the TATA box mutations were known to strongly affect transcription rate [4], [49].

We will focus on the proposed model for GAL10 found in [41] and perform our analysis to provide explanations on noise level invariance under TATA box mutations and to suggest other control schemes. The same model parameter values were used as found in [41]: The total number of the promoter (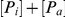) was assumed to be 1, and 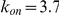, 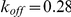, 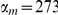, 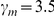, 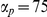, 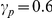 with the unit of all the parameters 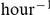, where the number of molecules is considered unitless.

**Figure 5:**
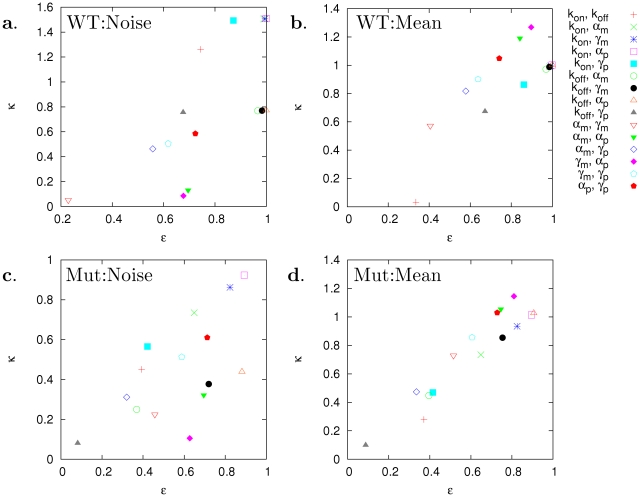
Efficiency and strength of orthogonal control in the Gal10 promoter expression (**Fig. 4a**). All possible two-parameter controls were considered. The efficiency 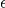 and strength 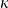 were computed by using Eqs. (6) and (7) with constraint tolerance 5% (see the Methods). (a) and (c): Among noise reduction control schemes, the most efficient and strongest control schemes were related to gene activation 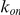. (b) and (d): Among mean-level reduction control schemes, the most efficient and strongest control schemes were related to translation 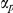.

We computed the CCs for the mean and noise levels for all the parameters for the wild-type promoter (Fig. 4c). The control coefficients with respect to 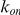 were found to be 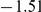 for the noise level and 0.07 for the mean level, showing that the noise level can be highly controllable by perturbing 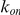 while the mean level cannot. The control coefficients with respect to 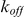 were 0.77 for the noise level and 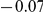 for the mean level, implying the same story as in the control case of 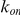. Thus, the mean level is not controllable for the two parameter control case (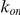, 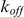) while the noise level is (Fig. 5a and b). The TATA box mutation experiments [41], however, show the control of (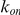, 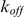) causes the change in the mean level but not in the noise level. We considered that this inconsistency arose from the fact that the control coefficients refer to sensitivity to infinitesimal parameter changes, while the TATA box mutations most likely correspond to finite parameter changes [41].

Thus, we computed again CCs for a TATA box mutation case, where the promoter deactivation was increased 225 times and activation 15 times. This control reduced the mean level by 50% without any significant change in the noise level (similar to the TATA box mutation, int1 in [41]) as shown in stochastic simulation results (Fig. 4e). The CCs for the mean level were significantly changed (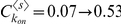; 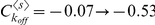), while those for the noise level were not (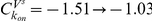; 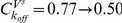). The significant change in the CCs for the mean level indicates that the mean level became controllable: The strength of the mean level control, 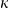, increased significantly (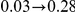) for the control scheme (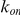, 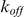), with a minor increase in the control efficiency 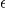 (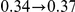) (Fig. 5b and d).

For both the wild type and the mutated cases, the computed control coefficients for noise levels satisfy their ratios, 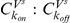, approximately to be 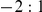 (Fig. 4c). This means that the noise level will not change when 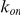 and 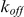 are perturbed infinitesimally by 1∶2 ratio, i.e., 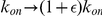; 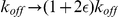 with 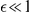, and for a finite perturbation, 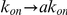 and 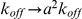 with a finite positive constant 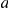 (see the Text S1). This is why the promoter deactivation and activation was perturbed by 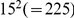 and 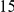 times, respectively. This ratio invariance in the TATA box mutations might be based on certain underlying biological mechanisms that are neglected in the simplistic two-state description of the promoter.

Based on the computed control strength and efficiency (Fig. 5), the best two-parameter control schemes were shown to be related to 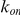 for noise control and to 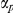 for mean level control. Stochastic simulations [47] were performed to verify the predicted orthogonal control methods and successfully showed that the noise/mean level was significantly reduced without changing the mean/noise level (Fig. 4d and e).

### Control of gene expression dynamics

**Figure 6:**
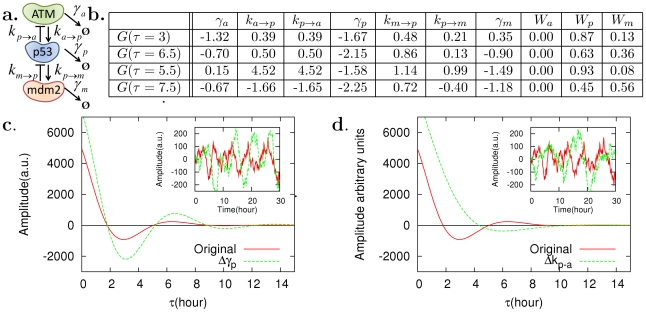
Control of p53 oscillations caused by DNA damage. (a) ATM protein kinases are activated in response to a DNA damage and phosphorylate p53, which activates the WIP1 gene that inhibits the ATM [68]. The phosphorylated p53 activates *mdm2* at the transcription level and Mdm2 binds to p53 with the Mdm2-p53 complex undergoing enhanced degradation. These negative feedback loops among ATM, p53, and Mdm2 cause sustained noisy oscillation at the p53 level [32], [51]. (b) Based on the model proposed by Geva-zatorsky et al. [32], control coefficients were computed. (c) Accordingly perturbation experiments were designed. Autocorrelation functions of p53 showed damped oscillations and their amplitudes were increased by decreasing the effective degradation rate 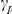 of p53 by 50%. (d) The oscillation period was increased by decreasing the inhibitory regulation of p53 on ATM 10 times. Refer to Text S1 for the details of the model. We note that the CCs for correlations also satisfy summation theorems (Text S1), indicating nontrivial correlation among the sensitivities.

We can also apply our analysis to control dynamics. Temporal noise correlations have been used to understand the topology of gene networks and their dynamical properties, such as *E. coli* CRP-GalS-GalE feedforward related to galactose metabolism [3], HIV Tat-mediated positive feedback [6], and cell damage response of p53-Mdm2 [32]. Thus, sensitivity analysis on the temporal correlation can provide a method for controlling the attributes of the dynamics. We consider the cell damage response of p53-Mdm2 and its stochastic model presented in [32] (Fig. 6a). The model describes the stochastic fluctuations in p53 and Mdm2 by using Langevin equations with Gaussian white noise (Text S1), and provided successful explanations on sustained noisy oscillations in p53 and Mdm2 under DNA damage [32]. We apply the CCs for the autocorrelation to control the amplitude and period of the oscillations.

The autocorrelation of p53 shows damped-oscillations (Fig. 6c and d), implying potential sustained noisy oscillations. Here it is aimed to increase the oscillation amplitude or period. First, consider amplitude controls. An amplitude increase can be reflected in the autocorrelation as an increased vertical separation between troughs and peaks. For such an increase, the computed CCs at 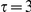 and 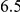 hr (corresponding to the trough and peak; Fig. 6c) need to be large same-sign values. This control does not belong to orthogonal control since both the trough and peak heights need to increase together, and can be mathematically described by using Eq. (3):
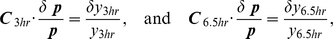
where both 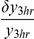 and 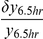 are real same-sign values with similar magnitude, and 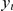 indicates the value of autocorrelation at time 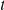.

We consider one-parameter controls, and then the inner products in the above equations become number products, indicating that 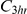 and 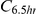 are real same sign values with the similar order of magnitude. This is well satisfied by the control coefficients corresponding to 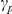. Thus, we decreased 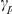 by 50% and this led to a visible increase in the p53 oscillation amplitude (Fig. 6c). Experimentally, p53 effective degradation, 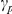, was reduced by introducing the small molecule Nutlin3 that inhibits p53 from binding to Mdm2 [50], [51] (the Mdm2-p53 complex shows enhanced degradation) and the oscillation amplitude was found to increase without affecting the period [51].

Second, consider period controls. The period increase causes the stretch-out of the autocorrelation in 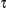-axis. This implies that the CCs at 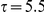 and 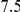 hr (corresponding to 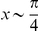 and 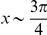 in 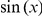, which decreases and increases when the sine function shifts to the right, respectively) need to be large opposite-sign values, respectively (Fig. 6b). Mathemtically, Eq. (3) is expressed as
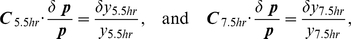
where 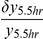 and 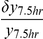 are real opposite-sign values with similar magnitude.

For one-parameter control, the above equations indicate that 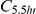 and 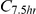 are real opposite-sign values with similar magnitude. Both the controls on 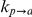 and 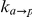 were found to be the best case. When one of these parameters was decreased to its 10% levels of the original value, a significant increase in the period was obtained (Fig. 6d). The decrease in 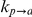 or 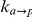 causes the ATM level to decrease and experimentally this can be achieved by decreasing 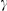-irradiation intensity [52]. (For cases without the irradiation, 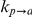 and 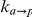 can be considered to vanish, resulting in a second-order linear model in [32].) Our analysis based on control coefficients showed successful control on noisy oscillation. This can serve as an important tool for analyzing the parameter dependence of stochastic dynamics, particularly when an analogous deterministic counterpart does not exist.

### Network structure and orthogonal control

**Figure 7:**
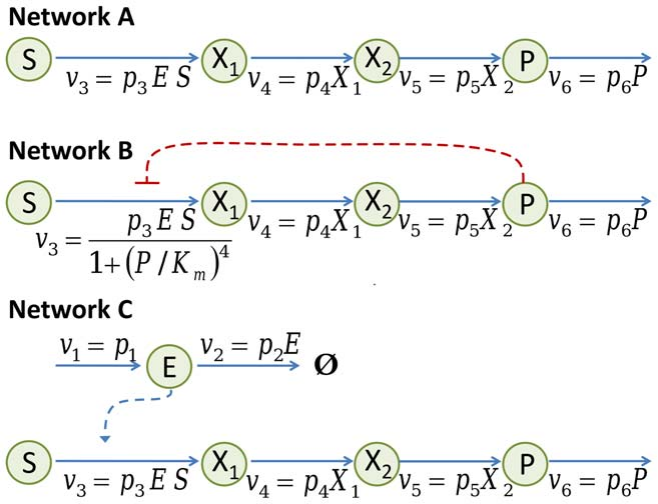
Linear metabolic pathways. For the network A and B, the enzyme level 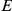 was fixed to 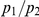. For the network C, the enzyme level was allowed to fluctuate due to its random synthesis and degradation. The parameter values: 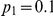 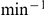, 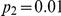 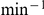, 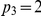 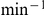, 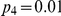 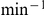, 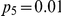 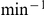, 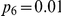 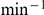, 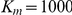. Here, we consider the number of molecules is dimensionless.

In this section, we will investigate the relationship between noise control and network structure. To show the applicability of our analysis to other systems, we will consider metabolic networks. It has been known that noise at enzyme levels causes metabolic flux to fluctuate and eventually to reduce the growth rate of host cells [16] due to nonlinearity in the system and noise propagation [40], [53] from the enzyme to the pathway. Here, we consider linear metabolic pathways (Fig. 7) and aim to reduce the noise level of the end product (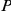 in Fig. 7) without altering its mean level. One of the enzymes (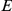) is considered explicitly and is used to supply extrinsic noise to the metabolic network. If such orthogonal noise reduction is achieved, the decrease in the growth rate that would have occurred due to the noise propagation can be suppressed.

Here, we show that feedback in the metabolic network and noise propagation [17], [40] from enzyme fluctuation [16] play important roles in enabling orthogonal control. We will limit control parameters to 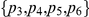 for ease of comparison between the original network and its variants.

First, consider the metabolic network under a constant enzyme level 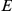 (located in the first step) and without any feedback (Network A in Fig. 7). It is known that at the stationary state, the probability distribution function of the whole system takes a product form and that inter-species covariance vanishes [23]–[25], resulting in the cancellation of the net effect of noise propagation [22]. This cancellation is related to network structure; the product form distribution was derived for mass-action systems (and some non-mass-action systems) [24] that satisfy the deficiency zero theorem [26]. This theorem is only dependent on the network structure. Furthermore, it was shown that each individual concentration distribution satisfies the Poisson distribution function [23], [24]. This indicates that the mean and noise levels are inversely related and that their control vectors are anti-parallel. Therefore, orthogonal control of the mean and noise levels cannot be achieved for any metabolites: 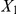, 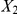, and 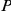. We verified this by computing the control vectors; for example, the control vectors for 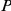 were obtained in the parameter space of 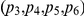:
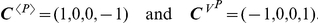
These vectors are anti-parallel, so control efficiency becomes zero. This fact implies that low control efficiency can be predicted by examining stoichiometry and topology.

Second, consider an end-product inhibition: negative feedback from 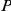 to the synthesis of 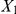 (Network B in Fig. 7). The covariances between metabolites were computed by using Eq. (8). The covariances between 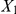 and 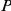 and between 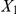 and 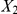 were found not to vanish. This implies that the stationary state does not take a product form distribution and that the Poisson distribution does not appear, either (the deficiency zero theorem [26] does not apply here, unless the mechanism of the feedback is expressed in terms of chemical reactions). Therefore, the control vectors will be no longer anti-parallel, providing the possibility of orthogonal control. The control vectors were computed:
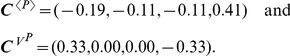
The control efficiency was significantly increased to 0.47, when compared with Network A.

Third, we consider the enzyme fluctuations in 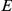 in the absence of negative feedback (Network C in Fig. 7). For this system, the product form distribution does not hold since the system is not weakly reversible (Text S1) [26] and the deficiency zero theorem does not apply. Noise originating from 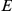 can be observed in metabolite fluctuations. The control vectors for 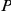 were computed:
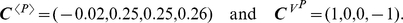
The control efficiency was further increased to 0.72, when compared with Network B. 

Finally, we allow both the noise propagation from 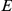 and the end-product inhibition. The control vectors were computed:
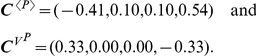
The control efficiency was decreased to 0.41, when compared with Network B and C. This is because the signs of the second and third elements of 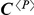 are opposite for Network B and C.

In the metabolic networks we consider, the application of extrinsic noise in 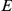 or the end-product inhibition significantly enhanced the control efficiency. This implies that in the case when orthogonal control cannot be performed with a high efficiency, perturbations in the network structure such as stoichiometry and topology can enhance the control efficiency. The result presented here, however, may not be directly applicable to gene regulatory networks, since gene expression processes occur in cascades of transcription and translation and thus they are not weakly reversible (similar to the case of the Network C).

### Iterative noise reduction

This section describes a computational protocol for iterative noise reduction. Since our analysis is based on differential sensitivities, infinitesimal perturbations can be continuously applied along the perturbation direction quantified by 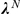, to achieve a finite-size perturbation. At the first level of approximation, the finite but small enough size of perturbations can be applied iteratively. We consider again the previous metabolic network models.

We performed the noise reduction control in the following sequence.

**Figure 8:**
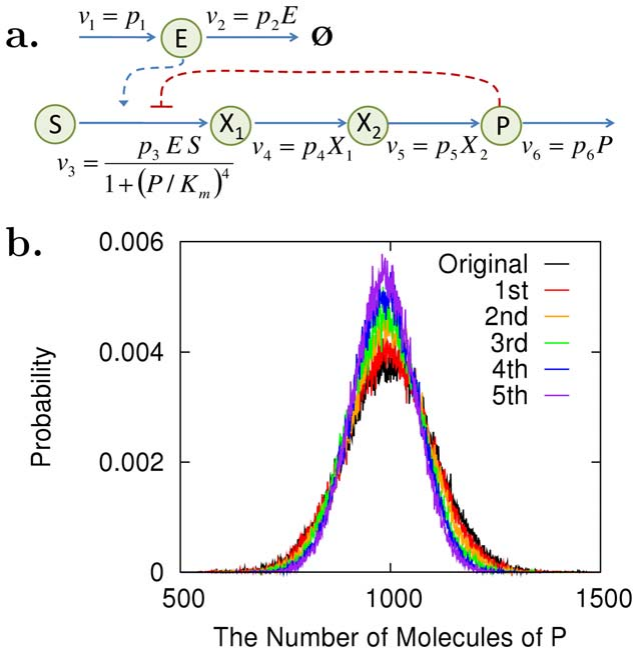
Noise control in a metabolic network under end-product inhibition. (a) The metabolic network is under stochastic fluctuations of an enzyme level 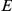. Other enzyme level fluctuations are neglected for simplicity. (b) Control analysis was applied to decrease the noise level of the end product, 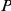, without changing its mean level. Iterative small perturbations reduced the noise level significantly with a minor change in the mean level, as shown by the change in the probability distribution functions of 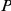. The original parameter values can be found in the caption of Fig. 7.

1. Compute control vectors for the mean and noise levels of 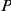: 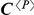 and 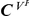.
2. Compute an orthogonal-control vector 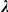 and normalize it to determine the direction of parameter perturbation. The normalized 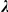 is denoted by 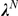. In Fig. 8, the original parameters (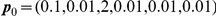 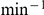) were perturbed along the direction of 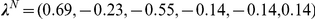.
3. Perturb the parameters by 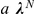, with 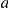 a proportionality constant that determines the size of the perturbation. Set the new value of 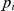 by 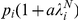 for all 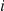.

We compare two cases with and without iteration. First, we performed a single large perturbation: 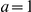. The noise was decreased by 36% (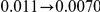), and the mean level by 11% (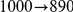). This non-negligible change in the mean level is due to the fact that the size of the perturbation is large enough that our analysis based on differential sensitivity becomes inaccurate. Second, we performed a series of small but finite perturbations: 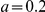 with 5 iterations by repeating the procedure (1)–(3). The noise level was significantly reduced by 50% (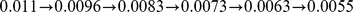), with a minor mean level decrease of 1.4% (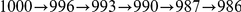), as shown by the change in the probability distribution functions of 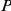 (Fig. 8C).

The protocol we describe is mathematically equivalent to a first order Euler approximation to find the parameter trajectory satisfying the control aim 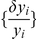, since the next parameter values are determined by the slope (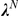) calculated at the current parameter values. The mean values deviate from the desired constant level on the order of magnitude of 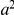: One Euler step updates parameters from 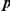 to 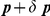, causing the mean value, here denoted by 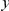, to change from 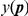 to 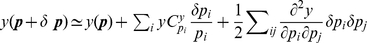, where the second term in the right hand side vanishes since 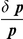 was set to be perpendicular to 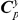. Therefore, the magnitude of the change is of the order of 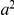.

## Discussion

In this paper we describe a systematic method for orthogonal control of noise and mean levels and provided its applications. In addition to these examples, our work can also be useful in synthetic biology.

In synthetic biology, biological organisms are engineered via design and construction of new useful biological functions that do not exist in nature. In synthetic gene regulatory networks (gene circuits), the signals are often considered the concentrations of transcription factors. Their copy numbers can be so low that their fluctuations are significant, meaning that the signals can be very noisy [54]. This causes cell-to-cell variability in gene expression levels and potentially their related phenotypes at the population and individual levels. In addition, the noise, both extrinsic and intrinsic, can propagate through a synthetic network [17], possibly preventing the predictable modular construction of circuits. From an engineering perspective, gene circuits have been designed and constructed based on the concept of modularity [33], [34], [55]–[60], to ensure predictable behavior when combining modular circuits. The reliability and predictability can be enhanced via simultaneous control of mean and noise levels by increasing signal-to-noise ratios and by suppressing unwanted noise propagation.

Noise control can also be used to improve gene circuit function. The properties of gene circuit components such as input-output responses can be engineered by exploiting noise. For example, noise can improve the sensitivity in a system response with respect to an input change via stochastic focusing [40], [53]. The noise can also help input signals be reliably transferred to output signals at a certain optimal level of intrinsic or input noise via stochastic resonance [61], [62]. These beneficial effects can be readily realized when the noise and mean levels can be independently controlled to their optimizing values.

For the p53 study, a frequency-domain analysis can be performed as an alternative approach. We can apply a Fourier transformation on Eq. (9), obtain its power spectral density, and compute control coefficients for the spectral density. The magnitude of the main spectral peak can be examined to quantify the oscillation amplitude, and the frequency corresponding to the main spectral peak can be used to determine the oscillation period. The reason that an autocorrelation function was used instead of its Fourier transform was that the numerical computation of the autocorrelation and its corresponding control coefficients can be performed without matrix inversion. Thus, it is computationally more efficient compared to using the spectral density, although control schemes for changing the period and amplitude might be more complex.

Our analysis is based on sensitivity to infinitesimal parameter changes and this was the reason that for the PHO5 promoter study the control parameters changed significantly depending on the specific parameter values, where the system responses to the parameter changes became highly nonlinear. Our approach can be used, however, as a first level of approximation for such cases, although the control schemes may not necessarily be the best ones. A global picture of controllability can be obtained by computing the sensitivities for various parameter values and determining the landscape of the sensitivities over the parameter space. If the landscape is flat, the proposed analysis can be applied to finite-size perturbations.

Our analysis is also based on the linear noise approximation [20]. The validity of this approximation needs to be verified on a case-by-case basis: The approximation depends on how large the noise levels in concentrations are compared to the (quasi-)linear region of the non-linear reaction rate functions. The strength of the noise level depends on how noise from the upstream network is propagated into the non-linear reaction rate functions. This means that the validity of the linear noise approximation crucially depends on noise propagation and the upstream network as well as the downstream non-linearity of reaction rate functions. Therefore, the linear noise approximation needs to be tested on a case-by-case basis. For the test, one possibility would be to use computer simulation for an exhaustive search.

Our analysis method can be applied to more complex networks than the systems previously considered. For example, consider the experiment on yeast cells performed in [63], where the expression noise of a reporter protein was controlled via transcriptional negative feedback. The reporter gene expression showed highly-sigmoidal dose-response in the absence of feedback, but it was linearized with the introduction of the feedback [64], [65]. The linearized dose-response led to smaller fluctuations in the response, when the input dose is centered around the sigmoidal region. Our analysis may be applied, for example, to increase/decrease the region of the linear dose-response by computing control coefficients for the mean levels of the reporter at two or more different input doses (e.g., three doses: 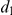, 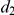, and 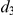) and by setting desired changes in the responses (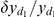, 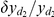, and 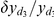), and by solving Eq (3) for 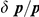:
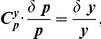
with 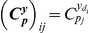 and 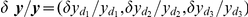. For more complex control, where multiple mean and noise levels are controlled simultaneously, Eq. (3) can be used again to identify control schemes computationally.

In summary, we have proposed a numerical analysis method for adjusting noise-related phenotype by controlling system parameters of mathematical models. The analysis quantifies which parameters need to be controlled by how much, with scaled non-dimensional values. In addition, we proposed how to improve control efficiency by changing network structure when control efficiency is weak. We have shown that MCA-like summation theorems exist and that the analysis can be applied to stochastic biological systems such as gene regulatory and metabolic networks and not only for statics but also for dynamics.

## Methods

### Computation of noise levels and autocorrelations

We consider stochastic reaction systems described as continuous time Markov processes. Stochastic fluctuations in concentrations caused by random reaction events are assumed to be small enough that the reaction law can be linearized with respect to the mean values for the study of the fluctuations. Such assumption is called the linear noise approximation [20]. Under this approximation, the covariance matrix 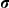 can be computed by solving the Lyapunov equation (also known as the fluctuation dissipation relationship [17], [66]):
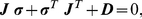
(8)with 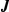 the Jacobian matrix and 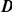 the diffusion matrix [17]. We compute noise levels (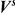) from 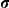:
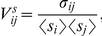
where 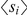 is the temporal average concentration level of the 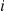-th species at the steady state. The autocorrelations 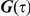 are defined as
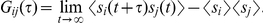
The autocorrelations can be computed by solving the following ordinary equation [31]:
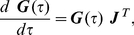
(9)for all 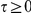, where 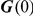 is equal to 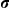. From Eq. (8) and (9), the noise levels and the autocorrelations can be computed numerically and analytically.

### Computation of stochastic control coefficients

The CCs for the noise levels and the autocorrelations can also be computed from Eq. (8) and (9). For mathematical simplicity, we will denote the matrix component 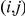 of 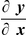 by 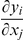, with 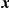 and 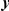 representing vectors. The Lyapunov equation (8) is invariant under parameter perturbations from one steady state to another corresponding to before and after the perturbation:
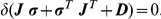
This can be expanded by using the chain rule:
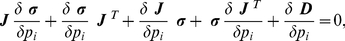
(10)where we have used 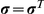 and 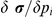 means the change in the concentration covariance matrix due to the change in 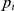, which defines an un-scaled CC for 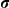. 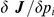 and 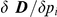 can be also expanded by applying the chain rule:
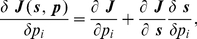
(11)

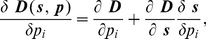
(12)where 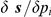 is an un-scaled control coefficient for mean concentration 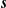 (for notation simplicity, instead of 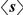). Under the linear noise approximation, concentration mean levels are obtained by using deterministic rate laws, neglecting noise propagation to the reaction rates [40]. Thus, the un-scaled CC can be obtained as in the deterministic MCA [27]–[29]:
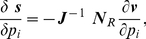
(13)where 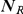 is a reduced stoichiometry matrix [67]. Equation (13) is substituted to Eqs. (11) and (12) and the resultant equations to Eq.(10), to numerically estimate the un-scaled CCs for 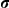, i.e., 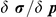.

Next, we obtain CCs for noise level. The noise level is defined as
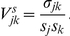
The un-scaled CCs for the noise level is expressed by applying the chain rule:
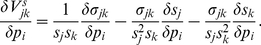
(14)By substituting Eq. (13) and the computed 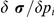 to Eq.(14), the un-scaled CCs for the noise level, i.e., 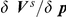 can be estimated and then converted to the scaled version:
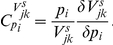
Next, we obtain CCs for autocorrelation functions. Equation (9) is invariant under parameter perturbations:
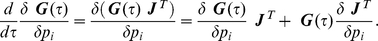
Since 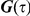 can be estimated by using Eq. (9) and 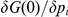 is equal to 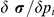, un-scaled CCs for 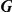 (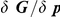) can be obtained by solving the above equation. This un-scaled CCs can be converted to the scaled version:
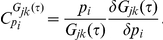
A MATHEMATICA file is provided for the estimation of CCs for noise levels in Text S2.

### Determination of 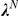


The Lagrange multiplier method will be used to obtain the direction 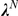 of parameter perturbation 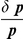 for orthogonal control of two system variables, 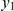 and 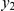, where 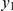 is increased but 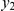 remains fixed: 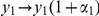 and 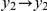. For non-degerate cases, 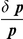 can be obtained by solving
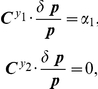
where 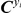 is a control vector for a variable 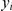. If the above equation is degenerate, the most optimal parameter perturbation needs to be identified. The solution can be considered optimal, if the net amount of parameter perturbations – the norm of 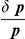 – is smallest among all possible solutions. We introduce Lagrange multipliers 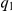 and 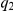 and the Lagrange function 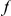:
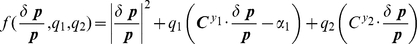
and solve
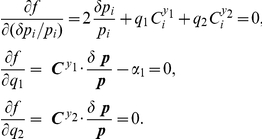
The solution of the first equation,
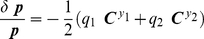
(15)is substituted in the second and third equations, which can be solved to obtain 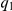 and 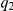:
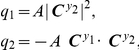
with
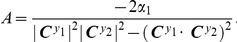
By substituting these equations to Eq. (15), we finally obtain the optimal paramter perturbation:
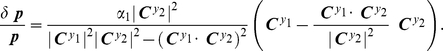
This perturbation, 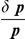, is normalized to obtain the direction of control, which is the same as that of the control vector expressed with Eq. (5) (since 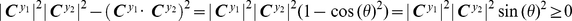, and 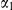 is negative if 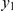 is considered as a noise level that is aimed to be reduced).

### Constraint tolerance

When the control vector for 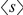 is small, 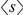 does not change significantly when the parameter perturbation is directed even toward that of the control vector. This means that the orthogonal control can be *effectively* performed over a much wider set of parameter perturbations, not just limited to the perpendicular plane to the control vector for 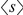. The norm of the control vector for 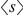 indicates the percentage change in 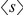 caused by a unit parameter perturbation directed along the control vector for 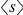. As the value of the norm decreases, the degeneracy of the orthogonal control increases.

**Figure 9:**
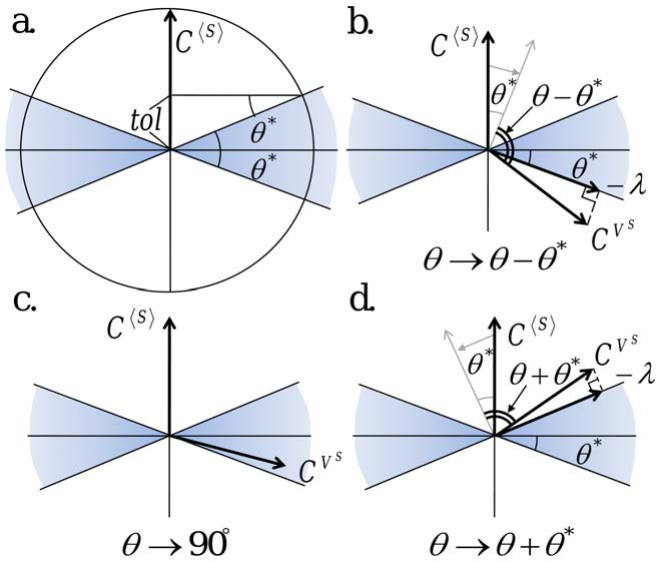
Control vector analysis for noise reduction with constraint tolerance. (a) When the vector length of 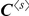 is small, the change in the mean level 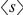 due to a parameter perturbation, although directed not perpendicular to 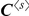, can be negligible within a certain tolerance level (tol). The parameter perturbation can be applied toward a direction that deviates from the perpendicular plane to 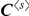 by an angle less than 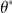 (defined in Eq (16)). (b–d) The angle between 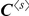 and 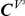 is modified differently depending on where 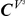 is placed.

Mathematically, when the mean value is allowed to change up to a certain tolerance level (tol) under a unit parameter perturbation, the perpendicular plane can be expanded up to a certain angle (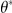) from the plane (Fig. 9a), which can be determined as follows:
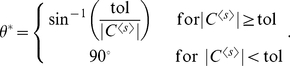
(16)This expanded perpendicular space (colored in Fig. 9) means that the control efficiency 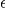 and strength 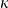 need to be re-defined: The control vector for the noise level is projected on the expanded perpendicular space, and for the most efficient control, projected on the closest one. Thus, the control efficiency and strength are re-defined by replacing the angle 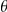 to the minimal angle from 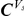 to the expanded perpendicular space (see Fig. 9b–d):
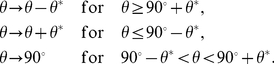
(17)


## Supporting Information

Text S1 In this document, control efficiency and strength are shown to change depending on the level of constraint tolerance for the yeast GAL10 promoter. The Lagevin model for the ATM-p53-mdm2 system is described in detail. Summation theorem for auto-correlation functions is derived. Jocobian and diffusion matrices are obtained for both the two-state model (HIV and GAL10) and the Langevin model (ATM-p53-mdm2). Brief discussion on why Network C in Fig. 7 is not weakly reversible is provided. Lastly, relationship between infinitesimal and finite perturbations in system parameters is discussed.
(PDF)

Text S2 This document provides a MATHEMATICA file for the estimation of CCs for noise levels.
(PDF)
