# Supplementary material for: Adjusting Phenotypes by Noise Control
Source: PLoS Comput Biol. 2012 Jan 12;8(1):e1002344. doi: 10.1371/journal.pcbi.1002344 (PMC3257291; doi:10.1371/journal.pcbi.1002344)
Supplement: Text S1 — In this document, control efficiency and strength are shown to change depending on the level of constraint tolerance for the yeast GAL10 promoter. The Lagevin model for the ATM-p53-mdm2 system is described in detail. Summation theorem for auto-correlation functions is derived. Jocobian and diffusion matrices are obtained for both the two-state model (HIV and GAL10) and the Langevin model (ATM-p53-mdm2). Brief discussion on why Network C in Fig. 7 is not weakly reversible is provided. Lastly, relationship between infinitesimal and finite perturbations in system parameters is discussed. (PDF) [file pcbi.1002344.s001.pdf]

# Supplementary Information

## Orthogonal noise control of the yeast GAL10 promoter

We consider the two-state model for the GAL10 promoter that was described in the main manuscript and compute control efficiency and strength for all possible two parameter control schemes. Here we aim to show the importance of introducing a tolerance level by showing that the control efficiency and strength can change significantly depending on the tolerance level.

For the case of control that the noise level is changed with the mean level fixed, the tolerance level (tol) is applied to the mean level. We examined the two cases: tol=0 and tol=5% for the wild-type. Among the two parameter control schemes, the one related to $(k_{on}, k_{off})$ was very dependent on the value of the tolerance level since the corresponding control coefficients for the mean value were very small ($C^{\langle s \rangle}_{k_{on}} = 0.07$ and $C^{\langle s \rangle}_{k_{off}} = -0.07$).

For the case of control that the mean level is changed with the noise level fixed, the tolerance level (tol) is applied to the noise level. The control schemes related to $\alpha_m$, $\gamma_m$, and $\alpha_p$ were significantly dependent on the tolerance level for the wild-type. This is because the control coefficients for the noise level with respect to these parameters were all small ($C^{V^s}_{\alpha_m} = -0.19$, $C^{V^s}_{\gamma_m} = 0.13$, and $C^{V^s}_{\alpha_p} = -0.01$).

## Model for ATM-p53-mdm2 oscillations

ATM-p53-mdm2 systems show sustained noisy oscillations, which were shown to be successfully modeled by the following linear Langevin equations [1]:

$$\begin{aligned}
\frac{da}{dt} &= k_{p\to a}p - \gamma_a a + \xi_a \\
\frac{dp}{dt} &= k_{a\to p}a + k_{m\to p}m - \gamma_p p + \xi_p \\
\frac{dm}{dt} &= k_{p\to m}p - \gamma_m m + \xi_m,
\end{aligned} \tag{S1}$$

where $a$, $p$, and $m$ denote the oscillation components of the concentrations of ATM, p53, and mdm2, respectively, and $\xi_a$, $\xi_p$, and $\xi_m$ are the independent Gaussian white noise, satisfying

$$\langle \xi_a(t)\xi_a(t') \rangle = W_a \delta(t - t')$$

and the same relationships for the other noise terms $\xi_p$ and $\xi_m$ with different noise strength $W_p$ and $W_m$. The parameter values are shown in Table S1.

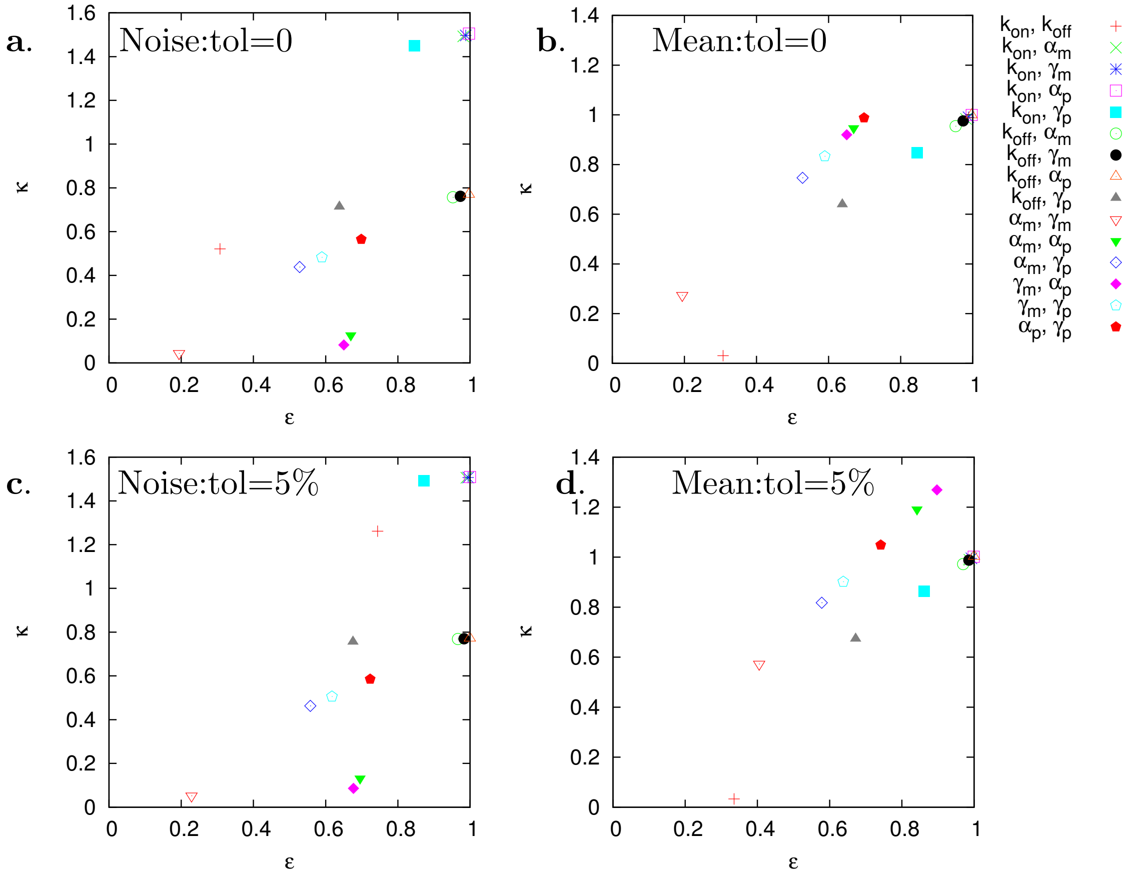


**Figure S1.** Control efficiency and strength for the wild-type GAL10 promoter for different tolerance levels (tol=0 and 5%). All possible two-parameter control schemes are considered. (a) and (c): Orthogonal noise level control. (b) and (d): Orthogonal mean level control

## Summation theorem for auto-correlation functions

We derived summation theorems among control coefficients for concentration mean and noise levels (coefficients of variation and even higher order moments) [2] that is analogous to those found in the metabolic control analysis. In this section, we derive another summation theorem regarding to control coefficients for auto-correlation functions.

We consider a system that is described by the master equation. The reaction rate functions can be arbitrary and the global proportionality constants are chosen for perturbations. For example, in the Michaelis-Menten-type rate law:

$$v(s) = v_{max} s/(s + K_M),$$

$v_{max}$ is our choice of parameter but the Michaelis-Menten constant $K_M$ is not. For more detailed discussion,

| $\gamma_a$ | $k_{a\to p}$ | $k_{p\to a}$ | $\gamma_p$ | $k_{m\to p}$ | $k_{p\to m}$ | $\gamma_m$ | $W_a$ | $W_p$ | $W_m$ |
|---|---|---|---|---|---|---|---|---|---|
| 60 ln(2)/100 | -0.65 | 1 | 60 ln(2)/75 | -0.55 | 0.29 | 60 ln(2)/144 | 1400 | 5000 | 10000 |

**Table S1.** Parameter values for the ATM-p53-mdm2 system. The unit of all the parameters is hour$^{-1}$. The values are taken from [1].

we refer to [2]. The set of the parameters will be denoted by $\boldsymbol{p}$. If there are $n$ reactions, the number of the parameters (the dimension of $\boldsymbol{p}$) is $n$.

Before the derivation of the summation theorem, we note that the rescaling all the parameter $\boldsymbol{p}$ to $\alpha\boldsymbol{p}$ means that all the reactions are increased by the factor of $\alpha$. This can be alternatively viewed as the unit of time is decreased by the factor of $\alpha$ [2–5]. This equivalence leads to the following relationship:

$$G(\tau, \alpha\boldsymbol{p}) = G(\alpha\tau, \boldsymbol{p}).$$

Specifically, we can obtain the above relationship as follows. The function $G(\tau, \alpha\boldsymbol{p})$ is defined as

$$G(\tau, \alpha\boldsymbol{p}) \equiv \langle X(t+\tau)X(t)\rangle - \langle X\rangle^2,$$

where the angle bracket means the average over time at the stationary state. With the rescaling in $\boldsymbol{p}$, the above equation becomes

$$G(\tau, \alpha\boldsymbol{p}) = \langle X(\alpha(t+\tau))X(\alpha t)\rangle - \langle X\rangle^2 = \langle X(\alpha t + \alpha\tau)X(\alpha t)\rangle - \langle X\rangle^2 = G(\alpha\tau, \boldsymbol{p}). \tag{S2}$$

By using this relationship, we can obtain the summation theorem as follows. First, an infinitesimal change in the autocorrelation function can be described by the sum of the change due to each individual parameter change by applying the chain rule:

$$\frac{\delta G(\tau,p)}{G(\tau)} = \sum_{i=1}^{n} \frac{1}{G}\frac{\delta G}{\delta p_i}\delta p_i = \sum_{i=1}^{n}\left(\frac{p_i}{G}\frac{\delta G}{\delta p_i}\right)\frac{\delta p_i}{p_i} = \sum_{i=1}^{n} C_{p_i}^G \frac{\delta p_i}{p_i} = (\alpha-1)\sum_{i=1}^{n} C_{p_i}^G \tag{S3}$$

Second, the infinitesimal change in the autocorrelation can also be described, by using Eq. (S2), as

$$\frac{\delta G(\tau,p)}{G(\tau)} = \frac{G(\tau,\alpha p) - G(\tau,p)}{G(\tau,p)} = \frac{G(\alpha\tau,p) - G(\tau,p)}{G(\tau,p)} = \big[(\alpha-1)\tau\big]\frac{1}{G(\tau,p)}\frac{\delta G(\tau,p)}{\delta\tau} = (\alpha-1)\frac{\delta\ln G(\tau,p)}{\delta\ln\tau}, \tag{S4}$$

where $\delta\tau = \alpha\tau - \tau = (\alpha-1)\tau$. From Eqs. (S3) and (S4), we obtain the summation theorem:

$$\sum_{i=1}^{n} C_{p_i}^G = \frac{\partial\ln G(\tau,p)}{\partial\ln\tau}.$$

## Jacobian and diffusion matrices

Our model system – a continuous time Markov process – is assumed to show stochastic fluctuations in state variables (here, concentrations) small enough that the fluctuations occur in linear portions of non-linear reaction rates. Under this assumption, the fluctuation statistics can be approximately described by linearizing the nonlinear reaction rates. This approximation is called the linear noise approximation [6]. The noise strength (concentration covariance) at the stationary state was shown to be computed by solving the Lyapunov equation [6] (also known as the fluctuation dissipation relationship [7, 8]):

$$\boldsymbol{J}\boldsymbol{\sigma} + \boldsymbol{\sigma}^T \boldsymbol{J}^T + \boldsymbol{D} = 0, \tag{S5}$$

where $\boldsymbol{\sigma}$ the covariance matrix, $\boldsymbol{J}$ the Jacobian matrix, and $\boldsymbol{D}$ the diffusion matrix.

When a system is governed by the master equation, $\boldsymbol{J}$ and $\boldsymbol{D}$ can be computed from the system's linearized reaction rates. However, when the master equation is not known, e.g., due to unknown source of noise, the system is often described by a phenomenological Langevin equation with Gaussian white noise, where the strength of noise is estimated via parameter fitting procedures. When fluctuations in concentrations are small enough that the Langevin equation can be linearized, the concentration covariance is described by the same (in mathematical structure) ordinary differentical equation that was derived from the master equation [9]. At the stationary state, the equation becomes the Lyapunov equation Eq. (S5) and the diffusion matrix is given by the estimated noise strength.

Consider the two-state gene expression model shown in Fig. 2b. This model system is fully described by the master equation. Under the linear noise approximation (the approximation becomes exact actually, since the model is described by linear reaction rate laws), $\boldsymbol{J}$ and $\boldsymbol{D}$ are given by

$$\boldsymbol{J} = \begin{pmatrix} -k_{off} - k_{on} & 0 & 0 \\ \alpha_m & -\gamma_m & 0 \\ 0 & \alpha_p & -\gamma_p \end{pmatrix},$$

$$\boldsymbol{D} = \begin{pmatrix} k_{on}[P_i] + k_{off}[P_a] & 0 & 0 \\ 0 & \alpha_m[P_a] + \gamma_m[mRNA] & 0 \\ 0 & 0 & \alpha_p[mRNA] + \gamma_p[Protein] \end{pmatrix},$$

where $[P_i]$, $[P_a]$, $[mRNA]$, and $[Protein]$ are concentrations at the stationary state, and the matrix index $(i, j)$ is defined as $i, j = 1$ corresponds to $P_a$; $i, j = 2$ to $mRNA$; and $i, j = 3$ to $Protein$. For example, both $\sigma_{12}$ and $\sigma_{21}$ represent covariance between $P_a$ and $mRNA$.

For the p53 oscillation model shown in Fig. 6a, the linear phenomenological Langevin equation (S1) was

used. $\boldsymbol{J}$ and $\boldsymbol{D}$ were given by

$$\boldsymbol{J} = \begin{pmatrix} -\gamma_a & k_{p\to a} & 0 \\ k_{a\to p} & -\gamma_p & k_{m\to p} \\ 0 & k_{p\to m} & -\gamma_m \end{pmatrix},$$

$$\boldsymbol{D} = \begin{pmatrix} W_a & 0 & 0 \\ 0 & W_p & 0 \\ 0 & 0 & W_m \end{pmatrix},$$

with the matrix index $(i,j)$ is defined as $i,j = 1$ corresponds to ATM; $i,j = 2$ to P53; and $i,j = 3$ to mdm2. The noise strength, $W_a$, $W_p$, and $W_m$ were estimated by parameter-fitting [1].

## Network C in Fig. 7 is not weakly reversible.

The network can be converted as follows:

$$\varnothing \underset{p_2 E}{\overset{p_1}{\rightleftharpoons}} E \xrightarrow{(p_3 S)E} X_1 + E,$$
$$X_1 \xrightarrow{p_4 X_1} X_2 \xrightarrow{p_5 X_2} P \xrightarrow{p_6 P} \varnothing \quad ,$$

where $S$ is a constant. This system is not weakly reversible; there is no directed arrow path, e.g., from the complex $X_1 + E$ to the complex $E$, from $X_2$ to $X_1$, and so on. The deficiency is, however, zero since there are six complexes $\{\varnothing, E, X_1 + E, X_1, X_2, P\}$, two linkage classes [10], and the rank of the network (the column rank of the stoichiometric matrix [10]) is 4.

## Relationship between infinitesimal and finite perturbations

Consider an infinitesimal perturbation in a parameter $p$ such as

$$p \to p(1+\epsilon),$$

with $\epsilon \ll 1$. The relative change in $p$ can be expressed as

$$\frac{\delta p}{p} = \delta \ln p = \epsilon.$$

The infinitesimal perturbation is applied iteratively in $N$ times to perturb the parameter in a finite amount:

$$\ln p_N - \ln p_0 = N\epsilon,$$

where $p_0$ and $p_N$ are the values of the parameter before any iteration performed and after the $N$-th iteration, respectively. Therefore, we obtain

$$p_N = p_0 \exp(N\epsilon).$$

When two parameters, $p$ and $q$, are perturbed infinitesimally with the ratio of 1:2:

$$p \rightarrow p(1+\epsilon), \text{ and } q \rightarrow q(1+2\epsilon),$$

with $\epsilon \ll 1$. After the $N$-th iteration of this perturbation, the final parameter values become:

$$p_N = p_0 \exp(N\epsilon), \text{ and } q_N = q_0 \exp(2N\epsilon).$$

Therefore, we obtain

$$\frac{q_N}{q_0} = \left(\frac{p_N}{p_0}\right)^2.$$

## References


1. Geva-Zatorsky N, Dekel E, Batchelor E, Lahav G, Alon U: **Fourier analysis and systems identification of the p53 feedback loop**. *Proc. Natl. Acad. Sci. U.S.A.* 2010, **107**(30):13550–13555.

2. Kim KH, Sauro HM: **Sensitivity summation theorems for stochastic biochemical reaction systems.** *Math. Biosci.* 2010, **226**(2):109–119.

3. Heinrich R, Rapoport SM, Rapoport TA: **Metabolic regulation and mathematical models**. *Prog. Biophys. Mol. Biol.* 1977, **32**:1–82.

4. Giersch C: **Control analysis of metabolic networks. 1. Homogeneous functions and the summation theorems for control coefficients.** *Eur. J. Biochem.* 1988, **174**(3):509–513.

5. Acerenza L, Sauro H, Kacser H: **Control analysis of time-dependent metabolic systems**. *J. Theor. Biol.* 1989, **137**(4):423–444.

6. Van Kampen NG: *Stochastic Processes in Physics and Chemistry.* North Holland, third edition 2001.

7. Kubo R: **The fluctuation-dissipation theorem.** *Rep. Prog. Phys.* 1966, **29**:255–284.

8. Paulsson J: **Summing up the noise in gene networks.** *Nature* 2004, **427**(6973):415–418.

9. Warren PB, Tănase-Nicola S, ten Wolde PR: **Exact results for noise power spectra in linear biochemical reaction networks.** *J. Chem. Phys.* 2006, **125**(14):144904.

10. Shinar G, Feinberg M: **Structural sources of robustness in biochemical reaction networks.** *Science (New York, N.Y.)* 2010, **327**(5971):1389–91.
