# Supplementary material for: Adjusting Phenotypes by Noise Control
Source: PLoS Comput Biol. 2012 Jan 12;8(1):e1002344. doi: 10.1371/journal.pcbi.1002344 (PMC3257291; doi:10.1371/journal.pcbi.1002344)
Supplement: Text S2 — This document provides a MATHEMATICA file for the estimation of CCs for noise levels. (PDF) [file pcbi.1002344.s002.pdf]

Supporting Information -- *Mathematica* Notebook file
Metabolic network with end-product inhibition

```
(* MODEL DEFINITION *)
(* NumReactions = Number of reactions *)
NumReactions = 6;
(* NumSpecies = Number of species *)
NumSpecies = 4;
(* Reaction rates *)
(* (#[[1]], #[[2]], #[[3]], #[[4]]) = (E, X1, X2, P) in Fig. 5 *)
(* Boundary species are set to 1 for simplicity -- For example, S in Fig.5. *)
v1 = p1 &;
v2 = p2 #[[1]] &;
v3 = p3 #[[1]] / (1 + (#[[4]] / km) ^4) &;
(*When the level of the enzyme E is fixed *)
(*v3=p3 10 / (1+(#[[4]]/km)^4) &;*)
v4 = p4 #[[2]] &;
v5 = p5 #[[3]] &;
v6 = p6 #[[4]] &;
(* Stoichiometry matrix *)
Nb = ( 1 -1  0  0  0  0
       0  0  1 -1  0  0
       0  0  0  1 -1  0
       0  0  0  0  1 -1 );
(* Initial condition -- Unit is min^-1. *)
pvalues = {p1 → 0.1, p2 → 0.01, p3 → 2, km → 1000, p4 → 0.01, p5 → 0.01, p6 → 0.01};
(*When the negative feedback from P to the synthesis of X1 is turned off*)
(*pvalues=
  {p1→ 0.1, p2→ 0.01, p3→2,km→100000000000, p4→0.01,p5→0.01, p6→0.01};*)
(* Estimation of CCs*)
Needs["PlotLegends`"];
Csigma = {};
Cs = {};
Results1 = {};
Results1det = {};
Results2 = {};
ResultConS0 = {};
ResultConS1 = {};
ResultConS2 = {};
ResultConSig0 = {};
ResultConSig1 = {};
ResultConSig2 = {};
Var = Table[si, {i, 1, NumSpecies}];
TestListRealPositive[x_] := (If[Length[x] == Length[Cases[x, _Real]],
```

```
    For[i = 1, i ≤ Length[x], i++, If[x[[i]] < 0 , Return[0]]]; Return[1],
    Return[0]]);
(* Concentration levels in the stationary state *)
(* If the levels are negative, they are neglected. *)
ssoldet =
 Select[NSolve[Nb.Table[vi[Var], {i, 1, NumReactions}] == 0 /. pvalues, Var],
  TestListRealPositive[Var /. #] == 1 &]
(* Check to see if there is one solution or not *)
(* If there exits only one solution,
proceed to estimate a concentration covariance matrix *)
(* partial v/partial p *)
vp = Table[D[vi[Var], pj], {i, 1, NumReactions}, {j, 1, NumReactions}];
(* partial v/partial s *)
vs = Table[D[vi[Var], sj], {i, 1, NumReactions}, {j, 1, NumSpecies}];
(* partial^2 v/partial s partial p *)
vsp = Table[D[vs[[i]][[j]], pk],
   {i, 1, NumReactions}, {j, 1, NumSpecies}, {k, 1, NumReactions}];
(* partial^2 v/partial s partial s *)
vss = Table[D[vs[[i]][[j]], sk],
   {i, 1, NumReactions}, {j, 1, NumSpecies}, {k, 1, NumSpecies}];

(* Jacobian matrix *)
J = Nb.vs /. pvalues /. ssoldet[[1]];
(* Covariance matrix *)
σ = Table[sigi,j, {i, 1, NumSpecies}, {j, 1, NumSpecies}];
(* Diffusion matrix -- Diff -- denoted by D in the manuscript *)
Λ = DiagonalMatrix[Table[vi[Var], {i, 1, NumReactions}]];
Diff = Nb. Λ. Transpose[Nb];
(* Covariance matrix, again : Numerical values are assigned. *)
Sig = Table[sigi,j, {i, 1, NumSpecies}, {j, 1, NumSpecies}] /.
  NSolve[J.σ + σ.Transpose[J] + Diff == 0 /. pvalues /. ssoldet, Flatten[σ]][[1]]
(* Control coefficients (scaled and unscaled) for concentration CV *)
(* sp = δs/δp = Unscaled concentration control coefficients *)
sp = -Inverse[J].Nb.vp /. pvalues /. ssoldet[[1]];
(* δJ/δp *)
Jp = Nb. vss. sp + Nb.vsp /. pvalues /. ssoldet[[1]];
(* δD/δp *)
Dp = Table[Sum[Nb[[i]][[m]] * (vp + vs.sp)[[m]][[k]] * Nb[[j]][[m]] /. pvalues /. ssoldet[[1]],
    {m, 1, NumReactions}], {i, 1, NumSpecies},
   {j, 1, NumSpecies}, {k, 1, NumReactions}];

CtrSigMatrix =
  Table[xmatrixi,j,k, {i, 1, NumSpecies}, {j, 1, NumSpecies}, {k, 1, NumReactions}];
(* δσ/δp = Unscaled control coefficients for concentration covariances *)
CtrSig = Solve[
```

```
    Table[Dp[[i]][[k]][[l]] + Sum[J[[i]][[j]] * xmatrix_{j,k,l} + J[[k]][[j]] * xmatrix_{i,j,l} +
          Jp[[i]][[j]][[l]] * Sig[[j]][[k]] + Jp[[k]][[j]][[l]] * Sig[[j]][[i]] , {j, 1, NumSpecies}],
        {i, 1, NumSpecies}, {k, 1, NumSpecies}, {l, 1, NumReactions}] == 0 /.
      pvalues /. ssoldet, Flatten[CtrSigMatrix]][[1]] ;
(* C^σ = Scaled control coefficients for concentration covariances *)
ScaledCtrSig = Table[CtrSigMatrix[[i]][[j]][[k]] / Sig[[i]][[j]] p_k /. pvalues /. ssoldet[[1]] /. CtrSig,
   {i, 1, NumSpecies}, {j, 1, NumSpecies}, {k, 1, NumReactions}];
(* δs/δp = Unscaled control coefficients for concentration mean values *)
CtrS = sp;
(* C^s = Scaled control coefficients for concentration mean values *)
ScaledCtrS = Table[CtrS[[i]][[k]] / s_i p_k /. pvalues /. ssoldet[[1]],
   {i, 1, NumSpecies}, {k, 1, NumReactions}];
(* Unscaled control coefficients for concentration CVs *)
(* Note: Coefficients of co-variation are not included in the following. *)
(* δV^s/δp *)
CtrVs =
  Table[CtrSigMatrix[[i]][[i]][[j]] / s_i^2 - 2 Sig[[i]][[i]] / s_i^3 CtrS[[i]][[j]] /. pvalues /. ssoldet[[1]] /.
    CtrSig, {i, 1, NumSpecies}, {j, 1, NumReactions}];
(* C^V *)
ScaledCtrVs =
  Table[(CtrSigMatrix[[i]][[i]][[j]] / s_i^2 - 2 Sig[[i]][[i]] / s_i^3 CtrS[[i]][[j]]) * s_i^2 p_j / Sig[[i]][[i]] /. pvalues /.
     ssoldet[[1]] /. CtrSig, {i, 1, NumSpecies}, {j, 1, NumReactions}];
(* Control vector for concentration CV of P *)
ScaledCtrVs[[4]]
(* Control vector for concentration mean value of P *)
ScaledCtrS[[4]]
(* Efficiency *)
Angle = ArcCos[ScaledCtrVs[[4]].ScaledCtrS[[4]] / (Norm[ScaledCtrVs[[4]]] Norm[ScaledCtrS[[4]]])] * 180/Pi;
Efficiency = Sin[Angle * Pi / 180]
(* Othogonal control vector for reducing the concentration CV *)
lambda = (-1) * (ScaledCtrVs[[4]] - ScaledCtrVs[[4]].ScaledCtrS[[4]] / Norm[ScaledCtrS[[4]]]^2 ScaledCtrS[[4]])
(* Normalized λ vector *)
lambdanorm = lambda / Norm[lambda]
(* Perturbation in parameters:
  The size of perturbation is proportional to the value of a. *)
(* Assign the perturbed values to new values of parameters. *)
```

```
a = 0.2; newvalues = {x1 → p1 (1 + a * lambdanorm[[1]]), x2 → p2 (1 + a * lambdanorm[[2]]),
   x3 → p3 (1 + a * lambdanorm[[3]]), x4 → p4 (1 + a * lambdanorm[[4]]),
   x5 → p5 (1 + a * lambdanorm[[5]]), x6 → p6 (1 + a * lambdanorm[[6]]), x7 → km} /. pvalues;
pvalues = newvalues /. {x1 → p1, x2 → p2, x3 → p3, x4 → p4, x5 → p5, x6 → p6, x7 → km}
```
